# Effect of 1 Year Krill Oil Supplementation on Cognitive Achievement of Dutch Adolescents: A Double-Blind Randomized Controlled Trial

**DOI:** 10.3390/nu11061230

**Published:** 2019-05-30

**Authors:** Inge S.M. van der Wurff, Clemens von Schacky, Trygve Bergeland, Roeslan Leontjevas, Maurice P. Zeegers, Jelle Jolles, Paul A. Kirschner, Renate H.M. de Groot

**Affiliations:** 1 Welten Institute, Research Centre for Learning, Teaching, and Technology, Open University of the Netherlands, 6419 AT Heerlen, The Netherlands; paul.kirschner@ou.nl (P.A.K.); renate.degroot@ou.nl (R.H.M.d.G.); 2 Omegametrix, 82 152 Martinsried, Germany; clemens.vonschacky@med.uni-muenchen.de; 3 Preventive Cardiology, Medical Clinic and Poli-Clinic I, Ludwig Maximilians-University Munich, 80336 Munich, Germany; 4 Aker BioMarine Antarctic AS, NO-1327 Lysaker, Norway; trygve.bergeland@getmail.no; 5 Faculty of Psychology and Educational Sciences, Open University of the Netherlands, 6419 AT Heerlen, The Netherlands; Roeslan.leontjevas@ou.nl; 6 Nutrition and Translational Research in Metabolism (School NUTRIM), Maastricht University, 6200 MD Maastricht, The Netherlands; m.zeegers@maastrichtuniversity.nl; 7 Care and Public Health Research Institute (School CAPHRI), Maastricht University, 6200 MD Maastricht, The Netherlands; 8 Section of Educational Neuroscience and LEARN! Research Institute, Faculty of Behavioural and Movement Sciences, Vrije Universiteit Amsterdam, 1081 BT Amsterdam, The Netherlands; j.jolles@vu.nl; 9 University of Oulu, FI-90014 Oulu, Finland

**Keywords:** docosahexaenoic acid (DHA), eicosapentaenoic acid (EPA), adolescents, cognition, omega-3 fatty acids, omega-3 Index, krill oil, randomized controlled trial (RCT), fatty acids

## Abstract

Long-chain polyunsaturated fatty acids (LCPUFA) are important for brain development and function, maybe especially during adolescence. Observational studies have demonstrated an association between fish consumption (a source of LCPUFA) and cognition in adolescents, but intervention trials are lacking. The goal of the current study was to investigate the effect of one year of krill oil (a source of LCPUFA) supplementation on the cognitive performance of adolescents with a low Omega-3 Index (O3I ≤ 5%). A double-blind, randomized, and placebo-controlled supplementation trial with repeated measurements (baseline (T0), three months (T1), six months (T2), and 12 months (T3)) in adolescents (267 randomized) was executed. Participants were randomized to 400 mg eicosapentaenoic acid (EPA) and docosahexaenoic acid (DHA) per day in Cohort I or placebo and 800 mg EPA + DHA per day in Cohort II or placebo. O3I was monitored by a finger prick at all time points. At T0, T2, and T3, participants executed a neurocognitive test battery. Covariate corrected mixed models were run with either condition (krill or placebo) or O3I as predictors. Krill oil supplementation led to a small but significant increase in mean O3I, but few participants increased to the intended O3I range (8–11%). There was no significant effect of supplementation on the neurocognitive tests, nor a relationship between O3I and neurocognitive test scores. The increase in O3I was small in most participants, probably due to non-compliance. Possibly the increase in O3I was too small to demonstrate an effect. More research on the influence of LCPUFAs on cognition in adolescents is needed.

## 1. Introduction

The influences of long-chain polyunsaturated fatty acids (LCPUFAs) on health and cognition have been studied extensively. Studies have focused on varying outcomes, ranging from cardiovascular health to diabetes, cognitive decline, and cognitive development [1,2,3,4]. The extensive research into LCPUFAs such as docosahexaenoic acid (DHA) and eicosapentaenoic acid (EPA) is not surprising, as they are involved in many processes such as neuronal membrane fluidity, signal transduction, blood-brain barrier integrity, and inflammation [5,6]. The possible positive influence of LCPUFAs such as DHA and EPA on brain functioning has led to many observational and experimental studies. However, these studies have mostly focused on infants, pregnant women, children, and the elderly (for review, see, among others, [7,8,9,10]), while LCPUFAs might be especially important in adolescents. 

Adolescence is characterized by profound brain development, with the prefrontal cortex especially continuing to mature into the late twenties [11,12]. In this period of brain development, the basis is laid for executive functions (e.g., shifting, updating, and short-term memory), among others. Optimal development of the prefrontal cortex is very important, as the executive functions have been related to academic achievements [13]. Considering brain development during adolescence and the important roles of LCPUFA in the brain, it is important to take the role of omega-3 LCPUFA into account. Moreover, the prefrontal cortex is very rich in DHA [14], and an earlier study with DHA supplementation in children aged 8–10 years showed higher functional activity in the prefrontal cortex [15]. 

Though a positive effect of LCPUFA on adolescent brain and cognition seems likely based on biological mechanisms, LCPUFA supplementation studies on cognition in healthy adolescents are, to the best of our knowledge, lacking. There are three observational studies in adolescents that show a positive association between fish consumption (an important source of LCPUFA) and cognition and/or school performance [16,17,18].

All in all, observational studies and biological mechanisms point to a possible positive relationship between LCPUFAs and cognitive performance in adolescents. We aimed to investigate the causal relationship between LCPUFA supplementation from krill oil and cognitive performance in adolescents in the second year of lower general secondary education (LGSE) who have a low Omega-3 Index (O3I). The goal was to increase the O3I of participants in the active treatment group to 8–11%. This target O3I was an estimate based on the O3I associated with the lowest mortality risk in coronary heart disease [19]. At the time the intervention started, the cardiovascular O3I target was the only one available. However, many of the mechanisms via which omega-3 fatty acids might be beneficial for the heart are also mechanisms via which it might be beneficial for the brain, i.e., anti-inflammatory properties and increased blood flow [6].

## 2. Materials and Methods

Food2Learn is registered at both the Netherlands Trial Register (NTR4082) and at Clinicaltrials.gov (NCT02240264). This study was conducted according to the guidelines laid down in the Declaration of Helsinki, and all procedures were approved by the Medical Ethical Committee of Atrium-Orbis-Zuyd Hospital, Heerlen, the Netherlands (NL45803.096.13). Written informed consent was obtained from all participants and at least one parent and/or caretaker. Below is a brief description of the procedure, design, and methods; more details have been reported previously [20]. 

### 2.1. Study Design

**Figure 1:**
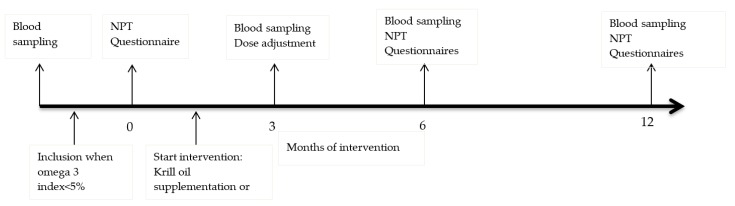
Timeline of study. NPT = neuropsychological tests, at all time points equal: Letter Digit Substitution Test, D2 test of attention, digit span forward and backward, Concept Shifting Test and Stroop test.

Food2Learn is a double-blind, randomized, placebo-controlled intervention trial with repeated measurements (baseline (T0), 3 months (T1), 6 months (T2), and 12 months (T3)) in which the effect of krill oil supplementation on, among others, the cognitive performance of second year high school students in the Netherlands was studied (see Figure 1). 

#### 2.1.1. Recruitment Procedure

Seventeen schools in the south of the Netherlands participated in the study. For the current study, all second year students of the LGSE theoretical learning pathway at the participating schools were approached in a classroom setting to participate. A research assistant explained the study and handed out information letters and informed consent forms. The students were asked to discuss the information with their parent(s) and/or guardian(s). When they wanted to participate, the informed consent form had to be signed by the adolescent as well as at least one parent and/or guardian. When the informed consent form was received, a finger prick was executed to determine the O3I in blood. As it was expected that any effect of krill oil supplementation would be more likely in participants with a low O3I, only those students with an O3I ≤ 5% were eligible to participate in the study. Together with the informed consent, parents and students filled out a short questionnaire with personal contact information, weight, height, and level of parental education.

#### 2.1.2. Participants

All students attending the LGSE theoretical learning pathway level at the participating school with an O3I ≤ 5% were eligible to participate. Exclusion criteria were an O3I > 5%, an allergy to fish or shell fish, or haemophilia. A total of 286 participants gave informed consent, of whom 267 were randomized. Participants were recruited in two cohorts: Cohort I (141 gave consent, 133 randomized) from November 2013 to February 2014 and Cohort II (147 gave consent, 134 randomized) from November 2014 to February 2015. Data were collected from February 2014 to April 2015 and from February 2015 to April 2016, respectively. 

#### 2.1.3. Randomization and Blinding

All participants received a participant number upon entering the study and were allocated to a condition by an independent researcher. Participants were stratified by sex, and an equal number of participants were allocated to the krill and placebo conditions; the group allocation sequence was computer generated. Both researchers at the site, as well as the participants and parents, were blind to the treatment condition. The packing of the boxes and placebo and krill oil capsules were, visually, exactly the same. Furthermore, capsules were coloured black, and a vanilla odour was added to ensure blinding. 

#### 2.1.4. Intervention

The intervention started after baseline neuropsychological testing. In Cohort I, participants were instructed to take four capsules (krill or placebo) daily with their dinner, the fattiest meal of the day [21]. Four krill oil capsules contained 260 mg EPA and 140 mg DHA, which was nearly the recommended amount of 450 mg DHA + EPA per day, as suggested by the Dutch Health Council [22]. After three months of supplementation, a personalized dose adjustment was planned to account for interpersonal difference in metabolism. However, at the three-month point, only 3 participants achieved the target O3I of 8–11%. Therefore, all participants were instructed to increase the daily dosage to eight capsules per day (both krill and placebo). Furthermore, it was decided that Cohort II would immediately start with eight capsules. Eight capsules of krill oil contained 520 mg EPA and 280 mg DHA per day. 

### 2.2. Data Collection

#### 2.2.1. Blood Analyses

Whole blood was obtained from a finger prick at T0, T1, T2, and T3 with an automated one-time use lancet and directly transferred to a filter paper (Whatman 903, General Electric, Frankfurt, Germany) pre-treated with a stabilizer. Filter papers were shipped immediately to Omegametrix, Martinsried, Germany, for analysis. Whole blood fatty acid compositions were analysed according to the HS-Omega-3 Index methodology as described previously [19,20,23]. 

#### 2.2.2. Cognitive Measurements

As the neuropsychological tests have been described in more detail in [20], a brief description follows. Five neuropsychological tests were administered at T0, T2, and T3. Three were administered in a small group setting (maximum 10 students) in a class room at school, namely The Letter digit substitution task (LDST), the D2 test of attention (D2), and the Digit Span backward and forward. After these group tests were administered, the Stroop Interference Test and the Concept Shifting Task were administered individually. The LDST is a measure for speed of information processing [24,25], the D2 test is used to measure selective attention [26], the Digit Span backward is a measure for working memory, the Digit Span forward is a measure for short term memory, the Stroop Interference Test is a measure for cognitive inhibition, and, lastly, the Concept Shifting Task is a measure for cognitive shifting [27]. 

#### 2.2.3. Other Measurements

After the neurocognitive tests, participants filled out a number of questionnaires. Among others, pubertal phase was assessed with the Pubertal Development Scale [28]. Students also filled out if they smoked cigarettes and, if so, how many, as well as if they drank alcohol and, if so, how often and how much. They also indicated whether they had a diagnosis which could influence learning (i.e., autism, dyslexia, or ADHD). At T2 and T3, questions were asked about compliance (how often did you forget the capsules?) and whether they experienced any side effects (open ended question). Lastly, at T3, students were asked to guess in which group they were allocated and to indicate how certain they were about their guess. 

### 2.3. Statistical Analyses

A sample size calculation was executed in R Mass (R Foundation, Vienna, Austria). To investigate the effect of the intervention on cognitive scores, mixed model analyses were executed in an R statistical environment (R studio version 3.2, RStudio, Inc., Boston, MA, USA), and all other analyses were executed with SPSS statistics version 24 (IBM, Chicago, IL, United States of America). 

#### 2.3.1. Sample Size

A sample size calculation was executed in R Mass software. Note that due to the uncertainties regarding the error variance and the intercept variation, we executed several calculations. Based on an effect size of d = 0.25 on the letter digit substitution task at six months and an effect size equal or 10% larger at 12 months, a drop-out rate of 25% per measurement moment (thus 43% in total), an error variation between 0.4 and 0.5, and an intercept variation of 0.3 to 0.5 with fixed effects, it was concluded that a sample of 183–285 participants would be sufficient to achieve a power of 0.8. 

#### 2.3.2. Group Comparisons, Treatment Guess and Adherence

Baseline comparisons on fatty acid concentrations, neurocognitive test scores, and participant characteristics (i.e., age, BMI, alcohol, sex, smoking, level of parental education, pubertal status, school, and cohort) were done with ANOVA analyses for the continuous variables and Chi square tests for the categorical variables. Participants in the krill oil group were compared with participants in the placebo group, those who completed the study were compared with those who dropped-out, and those who actively finished the study (i.e., taking placebo or krill oil) were compared with those who quit taking the capsules (both those that still participated in neuropsychological testing and those that quit completely). Moreover, fatty acid concentrations measured in blood at all time points in participants in the krill oil group were compared with fatty acid concentrations measured in blood in participants in the placebo group with an ANOVA. The treatment guess was compared for those in the krill oil group with the treatment guess in those in the placebo group with a Chi Square test. 

Students were asked to return capsules which they did not take as a measure of adherence. As an additional measure of adherence, the average increase in O3I between T0 and T2 and between T0 and T3 were studied. Moreover, the number of participants who had a decrease in O3I, had an increase up to 2.5%, and had an increase of more than 2.5% were noted.

#### 2.3.3. Imputing and Recoding Covariates

Data on drinking (units per week) and smoking (yes/no) were collected at T0 and at T3 and imputed for T2. An average score between 0 and 12 months was used for drinking, and a cut-off score of 0.5 cigarette a week was used to code yes/no for smoking (>0.5 as yes). Level of parental education was coded as low (vocational education and training and below) and high (university of applied sciences and higher). 

#### 2.3.4. Cognitive Measurements

Intention-to-treat analyses were conducted using linear mixed models that accounted for repeated measurements in subjects. Models allowed a comparison between groups (intervention versus control condition) and within groups (baseline data compared to intervention at other time points). Besides time trends (baseline as a reference) and treatment × time interactions, all estimates were adjusted for drinking behaviour, smoking behaviour, level of parental education, age at baseline, pubertal status at baseline, sex, body mass index, diagnosis related to learning, and cohort number.

Furthermore, moderation analyses for sex and, if relevant, sub group analyses for males and females were executed. 

Secondary analyses were executed with: (1) O3I as exploratory analyses and (2) only participants who completed the full study (i.e., had measurements on and T0, and T2, and T3). 

For the D2 total, D2 correct, LDST, Shifting, and Interference tests, the R package nlme with standard settings was used [29]. D2F1 (errors of omission on D2) and D2F2 (errors of commission) had skewed distributions and, therefore, the generalized linear mixed-effects (Poisson) model option and the bootstrapping method were used to determine the 95% confidence interval in the package lme4 [30]. Lastly, Digit Span data were analysed with the package ordinal with the function for cumulative link mixed models [31]. In these ordinal analyses, one quadrature point and flexible thresholds were used. In all cases, a *p* < 0.05 was considered to be statistically significant. 

## 3. Results

**Figure 2:**
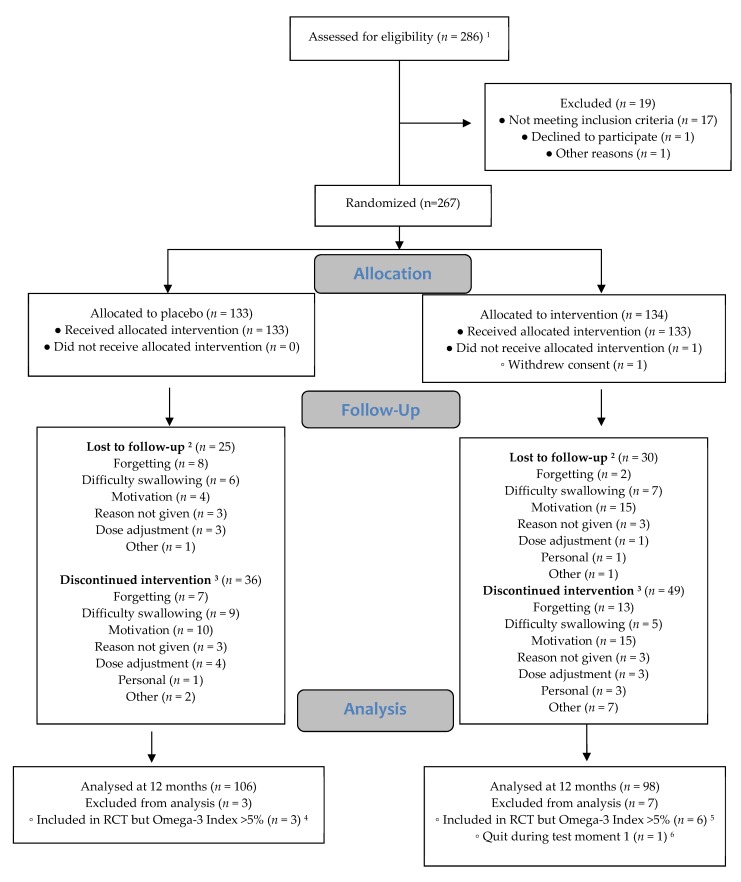
Flow chart. Flow chart adapted from Consort Guidelines [32] ^1^ Two additional participants provided consent, but then withdrew their consent again before blood sampling, these two participants are not included in this flowchart ^2^ Participants whom quite taking capsules and quite participation in testing. ^3^ Participants whom quite taking capsules but did participate in testing. ^4^ Three participants with an O3I >5% were included in the placebo group of these one was lost to follow up (reason forgetting), one discontinued the intervention (reason dose adjustment) and one finished the intervention, they were all excluded from the analyses. ^5^ Six participants with an O3I >5% were included in the intervention group of these one was lost to follow up (reason motivation), two discontinued the intervention (reason motivation/ personal) and three finished the intervention, they were all excluded from the analyses.^6^ This person is noted under lost to follow-up (reason other).

A total of 288 students provided informed consent, 19 did not meet inclusion criteria, one declined participation, and one was not included due to other reasons, yielding 267 respondents who were randomized into the study. Due to logistic reasons, nine participants with a baseline O3I > 5% were not excluded before the start of the trial. These participants were excluded from data analyses. One participant quit during baseline testing and was excluded from analyses. Thus, data were available for 257 participants for at least one time point. During the study, 52 students (20.3%) withdrew completely from the study, and 82 (32%) stopped active participation (i.e., they were tested, but did not take capsules, see Figure 2). Baseline characteristics for the placebo and the krill oil group can be found in Table 1, Table 2, and Table 3 (for characteristics separated by cohort see Supplementary Tables S1, S2, and S3). There were slightly more girls in the krill oil group than boys compared to the placebo group ((54 girls in the krill oil group versus 79 girls in the placebo group; χ (1) = 8.24; *p* = 0.004). There were no other differences between the placebo and krill oil groups in participant characteristics (all *p* > 0.185) or baseline cognitive test scores (all *p* > 0.131). 

### 3.1. Fatty Acids Concentrations

Concentrations of fatty acids in blood can be found in Table 2. The intervention group had a significant higher EPA, DPA, and DHA concentrations, a significantly higher O3I (all *p* <0.001), and significant lower concentrations of arachidonic acid (AA) and Osbond acid (ObA (all *p* < 0.038)) compared to the placebo group at T1, T2, and T3. 

### 3.2. Treatment Guess

After the one year supplementation period, 65 participants (67%) in the placebo group and 44 participants (49.4%) in the krill oil group correctly guessed their original treatment allocation. The percentage that correctly guessed treatment allocation did differ significantly, with more participants in the placebo group correctly guessing their group allocation (χ^2^ = 5.908; *p* = 0.017). 

### 3.3. Drop-Out and Adherence

There was a baseline difference between those who finished the study completely with supplementation and those who quit taking capsules for school and cohort. In some schools, more children quit supplementation than in other schools (χ^2^ = 28.299, *p* = 0.029). Moreover, there was a difference in drop-out between cohorts; more students stopped taking capsules in Cohort II (χ^2^ (1) = 11.329, *p* = 0.001) compared to Cohort I. All other participant characteristics, including fatty acids and neurocognitive test scores, were not significantly different between the groups.

Furthermore, when we compared active participants (those who took capsules) with participants who only took part in neuropsychological testing (without taking capsules) and participants that quit completely, the same patterns were seen. There was a difference between cohorts (χ^2^ = 13.139, *p* = 0.001) and between pubertal status at baseline, with those participating without taking capsules having a slightly lower pubertal status (M = 3.27 versus M = 3.52 for active participants, and M = 3.66 for those who dropped-out completely; F (2234) = 3.978; *p* = 0.020). All other participant characteristics, including fatty acids and neurocognitive test scores, were not significantly different between the groups. 

Students that were taking krill oil at T2 had an average increase compared to T0, with an O3I of 2.02% (SD 1.58). Compared to T0, at T2, five (6.8%) of the active participants in the krill group had a decrease in their O3I, 44 (60.3%) had an increase between 0 and 2.5%, and 24 (32.9%) had an increase of >2.5%. Compared to T2, at T3, there was an average decrease in the O3I of 0.70% in active krill oil participants. When looking at the O3I at T3 compared to T2, 35 (70%) of the active participants in the krill oil group had a decrease in their O3I and were thus most likely non-adherent to the protocol, and 15 (30%) had an increase between 0.14% and 1.96%. 

Participants were asked to return capsules at the last test moment. Of the active participants (i.e., those taking capsules) at T3, 56 handed in capsules, and 37 counted the left-over number of capsules at home. On average, participants handed in or counted at 628.82 ± 395.23 left-over capsules. As such, on average, participants did not take the capsules 78.6 days of the approximately 180 days between T2 and T3. 

### 3.4. Neuropsychological Tests

The random intercept models with time moment (T0, and T2, and T3), condition (krill oil or placebo), the interaction term (time moment × condition), and covariates showed that group allocation (krill or placebo) did not predict the score on any of the neurocognitive tests (see Table 4 and Table 5 and the Supplementary Tables S4, S5, S6, S7, and S8). There was, however, a clear time effect (i.e., students improved over time) on most neurocognitive tests. 

Moreover, there was an interaction effect for condition × time moment T3 for the number of target stimuli processed on the D2 (D2 correct). This indicates that the increase in number of correctly processed stimuli between T2 and T3 was slightly higher in the placebo group compared to the krill oil group, namely 4.71 stimuli more. 

The random intercept with time moment (T0, and T2, and T3), O3I, and covariates did not show an effect of O3I on neurocognitive test scores either. There was, however, again a clear time effect (i.e., students improved over time) on most neurocognitive tests. 

All above analyses were run with all participants whether they had data available for one, two, or three time points. To ensure the accuracy of the data, a secondary exploratory analysis was executed with only those participants that had data available at all three test moments. The results of these analyses were similar to the results of the intention to treat analyses (see Supplementary Tables S3 and S6). 

### 3.5. Side Effects

At the end of T2 and T3, we asked whether the participants had experienced any side effects. The question was open ended. At T2, 27 participants indicated to have a side effect. For krill, these were: six times positive (i.e., better focus, better school performance), once more hay fever, once t stomach ache (participant found out (s)he was allergic to fish), once stomach ache and nausea, once nausea and throwing up, once stomach ache and tiredness, once dizziness, and once throat ache. For placebo, these were: eight times positive; twice tired; once stomach ache; once hunger swings, more nervous, and mood swings; once gaining weight; and once ‘a weird feeling in my stomach.’ At T3, there were 29 participants with side effects. For the krill oil group, the following were reported: nine times positive; once nauseas and better memory; once throat ache; once head ache, stomach ache, sleeplessness, and little hunger; once nauseas; once head ache; once fishy taste when burping; once skin rash; and once headache, throat ache, nausea, and stomach ache. For the placebo group: five times positive, once tiredness, once dry skin, once less hunger and less concentration, once gaining weight and bad breath, once head ache, once ‘getting ill from it,’ and once stomach ache. 

## 4. Discussion

This study did not show an effect of one year of krill oil supplementation on a number of cognitive tests in typically developing adolescents of the lower general secondary education level in the Netherlands with a low baseline O3I. Moreover, sensitivity analyses did not show an association between O3I and neurocognitive test scores. However, the goals of the study were that participants in the krill oil group would achieve a predetermined O3I of 8–11% and that two distinct groups with regard to O3I would be achieved (thus no or minimal overlap in O3I between the krill and placebo groups). Unfortunately, neither goal was achieved. At six months, the follow-up the average O3I in the krill oil group was 5.29% (SD 1.61), and, at 12 months, it was 4.86 (SD 1.43). This was an average increase of 1.58% and 1.15%, respectively, compared to baseline. Moreover, between six months and 12 months, there was actually in average decrease in O3I in participants in the krill oil group who were said to take the capsules. Furthermore, only three, 10, and two participants achieved the target O3I of >8% at T1, T2, and T3, respectively. Thus, the average O3I of participants in the krill oil group was, at all time points, below the intended range of 8–11%, and it seems likely that participants were non-adherent to the protocol. 

Mixed model analyses did not show an effect of krill oil supplementation on cognitive test scores or a relationship between O3I and cognitive measures in the whole sample. It did show an interaction effect between condition and time moment T3 for the number of correctly processed stimuli on the D2, indicating that the increase in the correctly processed stimuli between T2 and T3 was slightly higher in the placebo group compared to the krill oil group. However, this effect was rather small (4.67, the average increase in score between T2 and T3 for the whole sample was 17.78 points), the placebo group scored higher on the D2 correct at every time point (albeit not significantly), and no relationships in the analyses with the O3I were shown. It is therefore not considered to be a notable finding. 

The findings that one year of krill oil supplementation did not improve cognitive test scores or a relationship between O3I and cognitive measures are in contrast with many supplementation studies in children [32,33,34,35,36], although not all studies in children show effects on cognition [37,38,39,40,41,42,43]. McNamara and colleagues did find increased functional activation of the dorsolateral prefrontal cortex after eight weeks of DHA supplementation of boys eight-to-ten years, but this did not translate into a difference in the performance of tasks [15]. Bauer et al. found similar results in adults, with participants in the DHA supplementation group showing an increase in the functional activity in the right precentral gyrus but no effect on cognitive measures [44]. Thus, the fact that in this study shows no effect of krill oil supplementation on the scores of cognitive tests does not preclude that there was increased activity in some brain regions. It remains to be seen, however, what the real life avail would be of an increased brain activation when this does not lead to improved performance on the relatively short-term (up to one year). In a previous paper, we reported on the baseline data of the current study and showed a positive association between higher O3I and better performance on the LDST in the whole sample and less D2 errors of omission [45]. It is unclear as to why that relationship could not be replicated in the current sample. 

The small increase in the O3I is likely caused by the high number of students that quit taking capsules and the high non-adherence in active participants. We tried to increase adherence to the protocol by sending a daily text message and having motivational talks with participants. However, the study might just have been too long in duration (the main reason for dropping-out was loss of motivation) and the number of capsules the students had to take was too high (another important reason for dropping-out was inability to take capsules). Both the long study duration and high number of capsules have been suggested to influence the adherence and drop-out rates [46]. This reasoning is also supported by the low drop-out and high adherence rates in the study of Tamman and colleagues. They studied a similar population, but the study only lasted 16 weeks, and students only had to take two capsules a day. They reported an adherence of 88% and a drop-out of 4.5% [47]. It is, however, important to note that if LCPUFA supplementation is found to be beneficial, long-term daily high dose intake of capsules might be needed to achieve and sustain effects, which seems to be extra problematic in adolescents. 

The strength of the current study is that the O3I was measured in blood. As such, a reliable measure for adherence was available, and analyses could be executed based on blood levels. Moreover, participants were preselected based on a low O3I, as it can be expected that any effects of supplementation would be more pronounced in those with a low baseline O3I. 

The main limitation of the current study is the fact that there was a relative high drop-out rate and adherence difficulties. However, the blood values and cognitive test scores for the majority of those that quit taking capsules were available. Moreover, participants that quit taking capsules did not differ significantly from those that continued the study actively on any of the relevant baseline measurements, and, thus, selection bias seems unlikely. 

To summarize, the current study did not show an effect of one year of krill oil supplementation on the cognitive measures of adolescents attending LGSE with a low baseline O3I. However, due to adherence problems and drop-out rates, it cannot be concluded that a relationship between krill oil supplementation and cognition does not exist. Though we acknowledge that the current study suffers from several limitations, we do hope that our study can be used as a starting point for future studies and can be used to learn from and improve future studies. More studies on the influence of krill oil supplementation on cognition in adolescents are needed. Moreover, these studies should focus on achieving a set target O3I in the active group. 

## Figures and Tables

**Table 1:** Participant characteristics at baseline.

	Placebo (M ± SD or N (%))	N	Krill (M ± SD or N (%))	N	*p* ^4^
Age (years)	14.07 ± 0.48	130	14.15 ± 0.51	126	0.185
Male/female N (%)	51/79 (39/61)	130	72/54 (57/43)	126	**0.004**
Smoking ^1^ (no/yes)	117/12 (91/9)	129	113/12 (90/10)	125	0.935
BMI ^2^	20.13 ± 3.05	123	19.84 ± 2.96	116	0.455
Alcohol units per week ^3^	0.34 ± 1.16	129	0.61 ± 2.29	126	0.234
Level of parental education (low/high)	54/69	123	49/66	115	0.840

^1^ Smoking was defined as anybody who indicated to smoke more than 0 cigarettes per week; ^2^ BMI was calculated weight (kg)/ length (m)^2^; ^3^ alcohol units per week was operationalized as number of days per week that alcohol is consumed times units per consumption moment; ^4^ ANOVA was used for age, BMI, LPE, and alcohol units per week, Chi Square for smoking, and sex. Significant differences (*p* < 0.05) are noted in bold.

**Table 2:** Fatty acids in blood at different time points in intention-to-treat analysis.

	Baseline			3 Months			6 Months ^1^			12 Months ^2^		
Fatty acid (%wt/wt of total FA)	placebo (*n* 130) Mean ± SD	krill (*n* 126) Mean ± SD	*p*	placebo (*n* 124) Mean ± SD	Krill ^3^ (*n* 118) Mean ± SD	*p*	placebo (*n* 116) Mean ± SD	krill (*n* 108) Mean ± SD	*p*	placebo (*n* 104) Mean ± SD	krill (*n* 95) Mean ± SD	*p*
AA 20:4n-6	11.19 ± 1.36	11.12 ± 1.18	0.657	10.97 ± 1.18	10.26 ± 1.13	**<0.001**	11.02 ± 1.49	10.28 ± 1.41	**<0.001**	11.15 ± 1.30	10.72 ± 1.49	**0.029**
EPA 20:5n-3	0.38 ± 0.14	0.38 ± 0.15	0.860	0.43 ± 0.15	0.93 ± 0.58	**<0.001**	0.41 ± 0.14	0.95 ± 0.69	**<0.001**	0.40 ± 0.12	0.75 ± 0.58	**<0.001**
ObA 22:5n-6	0.43 ± 0.11	0.45 ± 0.10	0.154	0.41 ± 0.17	0.32 ± 0.12	**<0.001**	0.42 ± 0.09	0.32 ± 0.11	**<0.001**	0.38 ± 0.12	0.34 ± 0.13	**0.038**
DPA 22:5n-3	1.22 ± 0.20	1.22 ± 0.17	0.858	1.29 ± 0.23	1.58 ± 0.34	**<0.001**	1.30 ± 0.20	1.54 ± 0.35	**<0.001**	1.30 ± 0.19	1.47 ± 0.31	**<0.001**
DHA22:6n-3	2.60 ± 0.44	2.49 ± 0.46	0.055	2.61 ± 0.52	3.25 ± 0.73	**<0.001**	2.69 ± 0.53	3.40 ± 0.90	**<0.001**	2.72 ± 0.54	3.20 ± 0.84	**<0.001**
O3I	3.83 ± 0.54	3.71 ± 0.55	0.106	3.88 ± 0.64	5.10 ± 1.29	**<0.001**	3.95 ± 0.64	5.29 ± 1.61	**<0.001**	3.98 ± 0.63	4.86 ± 1.43	**<0.001**

AA = arachidonic acid, DHA = docosahexaenoic acid, DPA = docosapentaenoic acid, EPA = eicosapentaenoic acid, O3I = Omega-3 Index, ObA = Osbond acid. Significant findings (*p* < 0.05) are printed in bold. ^1^ Please note that two participants did participate in testing but did not have a blood sample available ^2^ Please note that five participants did participate in testing but did not have a blood sample available. ^3^ Note this includes both the participants from Cohort 1, who took 400mg EPA + DHA per day, and participants from Cohort 2, who took 800mg EPA + DHA per day; separated analyses can be found in the supplemental material.

**Table 3:** Scores on the cognitive tests at different test moments in intention-to-treat analysis.

	Baseline			6 Months			12 Months		
	Placebo Mean ± SD Range	Krill Mean ± SD Range	*p*	Placebo Mean ± SD Range	Krill Mean ± SD Range	*p*	Placebo Mean ± SD Range	Krill Mean ± SD Range	*p*
LDST (number)	35.02 ± 5.73 21–53	33.98 ± 5.22 15–49	0.131	37.56 ± 6.13 24–52	36.44 ± 6.46 12–50	0.184	39.79 ± 5.78 26–59	38.39 ± 5.81 25–53	0.085
D2 total (number)	421.42 ± 57.69 310–562	416.37 ± 55.92 294–287	0.478	468.88 ± 61.71 314–636	461.41 ± 57.68 308–607	0.350	506.08 ± 65.88 370–653	494.22 ± 57.00 352–619	0.175
D2 correct (number)	164.82 ± 24.34 103–232	162.71 ± 22.29 111–223	0.472	187.91 ± 28.47 134–279	184.87 ± 25.59 128–261	0.403	206.88 ± 34.09 151–291	201.29 ± 28.17 140–271	0.208
D2 error of commission (number)	11.97 ± 9.55 0–58	11.67 ± 12.11 0–109	0.824	10.0 ± 9.62 0–55	9.44 ± 7.81 0–40	0.631	9.14 ± 10.59 0–56	8.22 ± 6.90 0–37	0.469
D2 error of omission (number)	1.38 ± 1.72 0–9	1.22 ± 1.49 0–6	0.442	1.12 ± 1.74 0–10	1.06 ± 1.35 0–7	0.793	0.85 ± 1.64 0–10	0.68 ± 1.10 0–6	0.388
Shifting score (s)	11.69 ± 6.51 –1.27–37.81	11.79 ± 7.21 1.39–38.33	0.912	10.30 ± 5.59 –3.83–36.58	9.99 ± 7.06 –2.35–36.41	0.715	10.26 ± 5.69 0.99–27.18	9.98 ± 7.20 –8.21–40.01	0.760
Interferences score (s)	31.64 ± 8.74 16.69–67.90	30.51 ± 7.79 16.34–52.32	0.274	28.01 ± 6.60 14.56–68.40	27.83 ± 6.14 16.31–42.29	0.834	26.77 ± 6.76 14.19–59.31	25.92 ± 5.31 16.75–40.67	0.321
Digit Span Forward (digits)	5.55 ± 0.83 3–8	5.61 ± 0.89 3–8	0.547	5.37 ± 0.80 4–8	5.53 ± 1.05 3–8	0.211	5.68 ± 0.98 3–8	5.64 ± 0.90 3–8	0.783
Digit Span Backward (digits)	4.58 ± 0.98 2–7	4.52 ± 1.00 2–7	0.668	4.71 ± 0.81 2–7	4.69 ± 0.91 2–7	0.816	4.83 ± 0.96 3–7	4.65 ± 1.11 3–7	0.224

LDST = letter digit substitution task. Note that a lower score on D2F1, D2F2, Shifting and Interference is a better performance, for all other tests a higher score is a better performance.

**Table 4:** Multilevel analyses of cognitive test scores predicted by condition (intention-to-treat) and according to Omega-3 Index.

Outcome Variable	Predictor		Estimate (SE)	95%CI		Estimate (SE)	95%CI
D2-Total	Test moment	T2	48.40 (3.62)	**(41.39; 55.41)**	T2	47.90 (3.01)	**(42.06; 53.74)**
		T3	89.00 (3.83)	**(81.59; 96.41)**	T3	84.88 (3.07)	**(78.93; 90.84)**
	Condition	Krill	−7.99 (8.06)	(−23.62; 7.65)	O3I	−0.20 (1.63)	(−3.36; 2.95)
	Interaction	T2 × krill	−0.99 (5.28)	(−11.21; 9.23)			
		T3 × krill	−9.17 (5.47)	(−19.77; 1.43)			
D2-Correct	Test moment	T2	23.01 (1.55)	**(20.00; 26.02)**	T2	23.01 (1.30)	**(20.50; 25.53)**
		T3	43.53 (1.64)	**(40.35; 46.71)**	T3	41.63 (1.32)	**(39.07; 44.19)**
	Condition	Krill	−1.53 (3.70)	(−8.72; 5.66)	O3I	−0.49 (0.71)	(−1.85; 0.88)
	Interaction	T2 × krill	−0.63 (2.26)	(−5.01; 3.76)			
		T3 × krill	−4.71 (2.35)	**(−9.26; −0.17)**			
D2-F1	Test moment	T2	−0.13 (0.04)	**(−0.21; −0.04)**	T2	−0.14 (0.04)	**(−0.21; −0.06)**
		T3	−0.24 (0.05)	**(−0.34; −0.14)**	T3	−0.27 (0.04)	**(−0.34; −0.19)**
	Condition	Krill	−0.10 (0.10)	(−0.29; 0.11)	O3I	0.01 (0.02)	(−0.03; 0.05)
	Interaction	T2 × krill	−0.001 (0.06)	(−0.13; 0.11)			
		T3 × krill	−0.05 (0.07)	(−0.18; 0.10)			
D2-F2	Test moment	T2	−0.24 (0.13)	(−0.48; 0.01)	T2	−0.13 (0.10)	(−0.30; 0.06)
		T3	−0.53 (0.14)	**(−0.82; −0.28)**	T3	−0.56 (0.12)	**(−0.78; −0.34)**
	Condition	Krill	−0.23 (0.16)	(−0.55; 0.08)	O3I	−0.03 (0.05)	(−0.14; 0.06)
	Interaction	T2 × krill	0.17 (0.19)	(−0.18; 0.52)			
		T3 × krill	−0.04 (0.23)	(−0.45; 0.38)			
	Test moment	T2	2.48 (0.48)	**(1.55; 3.41)**	T2	2.42 (0.39)	**(1.66; 3.18)**
LDST		T3	4.74 (0.50)	**(3.76; 5.72)**	T3	4.56 (0.40)	**(3.78; 5.33)**
	Condition	Krill	−0.18 (0.81)	(−1.75; 1.39)	O3I	−0.07 (0.20)	(−0.46; 0.32)
	Interaction	T2 × krill	−0.23 (0.70)	(−1.59; 1.12)			
		T3 × krill	−0.32 (0.72)	(−1.71; 1.08)			
Shifting	Test moment	T2	−1.59 (0.81)	**(−3.16; −0.02)**	T2	−1.80 (0.64)	**(−3.04; −0.56)**
		T3	−1.23 (0.85)	(−2.87; 0.41)	T3	−1.87 (0.66)	**(−3.15; −0.60)**
	Condition	Krill	−0.004 (0.90)	(−1.75; 1.74)	O3I	0.06 (0.27)	(−0.47; 0.58)
	Interaction	T2 × krill	−0.31 (1.18)	(−2.61; 1.98)			
		T3 × krill	−1.48 (1.22)	(−3.85; 0.88)			
Interference	Test moment	T2	−4.01 (0.72)	**(−5.40; −2.62)**	T2	−3.45 (0.58)	**(−4.57; −2.32)**
		T3	−5.41 (0.75)	**(−6.87; −3.96)**	T3	−5.38 (0.59)	**(−6.53; −4.24)**
	Condition	Krill	−1.10 (0.97)	(−2.98; 0.77)	O3I	−0.01 (0.28)	(−0.55; 0.53)
	Interaction	T2 × krill	1.14 (1.05)	(−0.89; 3.16)			
		T3 × krill	−0.12 (1.08)	(−2.21; 1.97)			

D2 = D2 test of attention, D2 correct = number of target stimuli processed, D2F1 = errors of omission on D2, D2F2 = errors of commission on D2, LDST = letter digit substitution task, O3I = Omega-3 Index, T2 = test moment 6 months, T3 = test moment 12 months All models included test moment, condition (Krill/placebo) plus covariates (alcohol consumption, smoking behavior, age at baseline, puberty status at baseline, BMI, highest level of parental education, sex, cohort number, and diagnosis). Significant 95% CI are printed in bold. The model with condition also includes the time moment × condition interaction factor. Note that a lower score on D2F1, D2F2, Shifting, and Interference is a better performance. Significant findings (*p* < 0.05) are printed in bold.

**Table 5:** Multilevel analyses of Digit Span forward and Digit Span backward, predicted by either condition (intention-to-treat) or Omega-3 Index.

Outcome Variable	Predictor	Odds Ratio (SE)	95%CI		Odds Ratio (SE)	95%CI
**Digit Span forward**						
Test moment	T2	0.44 (1.32)	**(0.26; 0.76)**	T2	0.54 (1.25)	**(0.35; 0.84)**
	T3	1.15 (1.33)	(0.66; 2.02)	T3	1.16 (1.25)	(0.75; 1.80)
Condition ^1^	Krill	0.85 (1.41)	(0.43; 1.68)	O3I	1.08 (1.11)	(0.88; 1.32)
Interaction ^2^	T2 × krill	1.88 (1.50)	(0.85; 4.14)			
	T3 × krill	1.22 (1.51)	(0.54; 2.73)			
**Digit Span backward**						
Test moment	T2	1.38 (1.30)	(0.83; 2.30)	T2	1.49 (1.23)	**(1.00; 2.23)**
	T3	1.61 (1.32)	(0.93; 2.78)	T3	1.61 (1.24)	**(1.06; 2.46)**
Condition ^1^	Krill	0.85 (1.37)	(0.46; 1.58)	O3I	1.00 (1.10)	(0.83; 1.21)
Interaction ^2^	T2 × krill	1.15 (1.46)	(0.55; 2.42)			
	T3 × krill	0.98 (1.49)	(0.45; 2.13)			

O3I = Omega-3 Index, T2 = test moment 6 months, T3 = test moment 12 months. All models included test moment, condition (krill/placebo), or Omgea-3 Index plus covariates (alcohol consumption, smoking behavior, age at baseline, puberty status at baseline, BMI, highest level of parental education, sex, cohort number, and diagnosis). The model with condition also includes the time moment × condition interaction factor. Please note that for odds ratios significance is indicated by a 95% CI which does not contain 1, these are printed in bold. ^1^ The factor krill indicates the score of somebody in the krill oil group compared to the placebo group. ^2^ Interaction term of krill and time point.

## Supplementary Materials

The following are available online at https://www.mdpi.com/2072-6643/11/6/1230/s1, Table S1: Participant characteristics at baseline separate for cohort 1 and 2, Table S2: Fatty acids in blood at different time points intention to treat separate for cohort 1 and 2, Table S3: Multilevel analyses of cognitive test scores predicted by condition and Omega-3 Index, participant whom participated in all test moments, Table S4: Multilevel analyses of cognitive test scores predicted by condition (intention to treat) and according to Omega-3 Index for cohort 1, Table S5: Multilevel analyses of cognitive test scores predicted by condition (intention to treat) and according to Omega-3 Index for cohort 2, Table S6: Multilevel analyses of DSF and DSB predicted by condition and Omega-3 Index, participant whom participated in all test moments, Table S7: Multilevel analyses of digit span forward and digit span backward, predicted by either condition (intention to treat) or Omega-3 Index for Cohort 1, Table S8: Multilevel analyses of digit span forward and digit span backward, predicted by either condition (intention to treat) or Omega-3 Index for Cohort 2.
