# Supplementary material for: Effect of 1 Year Krill Oil Supplementation on Cognitive Achievement of Dutch Adolescents: A Double-Blind Randomized Controlled Trial
Source: Nutrients. 2019 May 30;11(6):1230. doi: 10.3390/nu11061230 (PMC6628105; doi:10.3390/nu11061230)
Supplement: Supplementary file 1 [file nutrients-11-01230-s001.pdf]

Supplementary Table S1: Participant characteristics at baseline separate for cohort 1 and 2

	Placebo	N	Krill	N	P[4]
Cohort 1					
Age (years)	14.09 ± 0.46	69	14.18 ± 0.54	56	0.297
Male/female N (%)	23/46	69	30/26	56	**0.023**
Smoking[1] (no/yes)	65/3	68	49/6	55	0.169
BMI	20.36 ± 2.96	63	20.13 ± 2.79	46	0.671
Alcohol units per week[2]	0.29 ± 0.77	68	0.85 ± 3.09	56	0.154
Level of parental education (low/high)	31/32	63	21/26	47	0.638
Cohort 2					
Age (years)	14.04 ± 0.51	61	14.12 ± 0.49	70	0.352
Male/female N (%)	28/33	61	42/28	70	0.107
Smoking[1] (no/yes)	52/9	61	64/6	70	0.268
BMI[2]	19.89 ± 3.15	60	19.65 ± 3.07	70	0.670
Alcohol units per week[3]	0.39 ± 1.48	61	0.41 ± 1.33	70	0.917
Level of parental education (low/high)	23/37	60	28/40	68	0.743

[1] Smoking was defined as anybody who indicated to smoke more than 0 cigarettes per week; [2] BMI was calculated weight (kg)/ length (m) [3]Alcohol units per week was operationalized as number of days per week that alcohol is consumed times units per consumption moment; [4]ANOVA was used for age, BMI, LPE and alcohol units per week, Chi Square for smoking, and sex. Significant differences ($p < 0.05$) are noted in bold.

Supplementary Table S2: Fatty acids in blood at different time points intention to treat separate for cohort 1 and 2.

Fatty acid (%wt/wt of total FA)	Baseline			3 months			6 months			12 months		
Cohort 1	Placebo Mean ± SD (n 69)	Krill Mean ± SD (n 56)	P	Placebo Mean ± SD (n 64)	Krill Mean ± SD (n 51)	P	Placebo Mean ± SD (n 60)	Krill Mean ± SD (n 48)	P	Placebo Mean ± SD (n 57)	Krill Mean ± SD (n 40)	P
AA 20:4n-6	11.33 ± 1.24	11.01 ± 0.96	0.117	11.02 ± 1.16	10.16 ± 0.95	**<0.001**	11.10 ± 1.55	9.99 ± 1.30	**<0.001**	11.03 ± 1.32	10.17 ± 1.40	**0.003**
EPA 20:5n-3	0.41 ± 0.21	0.42 ± 0.13	0.701	0.45 ± 0.15	0.86 ± 0.35	**<0.001**	0.42 ± 0.14	1.06 ± 0.71	**<0.001**	0.40 ± 0.13	0.79 ± 0.60	**<0.001**
ObA 22:5n-6	0.41 ± 0.09	0.42 ± 0.08	0.732	0.37 ± 0.09	0.27 ± 0.06	**<0.001**	0.42 ± 0.09	0.29 ± 0.10	**<0.001**	0.34 ± 0.12	0.30 ± 0.13	0.067
DPA 22:5n-3	1.21 ±0.18	1.22 ± 0.16	0.741	1.30 ± 0.19	1.54 ± 0.23	**<0.001**	1.30 ± 0.16	1.57 ± 0.36	**<0.001**	1.30 ± 0.19	1.50 ± 0.33	**<0.001**
DHA 22:6n-3	2.63 ± 0.40	2.49 ±0.52	0.088	2.72 ± 0.49	3.34 ± 0.64	**<0.001**	2.67 ±0.52	3.48 ± 0.95	**<0.001**	2.75 ± 0.54	3.41 ± 0.91	**<0.001**
O3I	3.90 ± 0.48	3.76 ±0.61	0.156	4.02 ± 0.62	5.12 ± 0.97	**<0.001**	3.95 ± 0.61	5.48 ± 1.67	**<0.001**	4.01 ± 0.62	5.13 ± 1.53	**<0.001**
Cohort 2	Placebo Mean ± SD (n 61)	Krill Mean ± SD (n 70)		Placebo Mean ± SD (n 60)	Krill Mean ± SD (n 67)	P	Placebo Mean ± SD (n 56)	Krill Mean ± SD (n 60)	P	Placebo Mean ± SD (n 47)	Krill Mean ± SD (n 55)	P
AA 20:4n-6	11.03 ± 1.47	11.21 ±1.33	0.477	10.92 ± 1.21	10.34 ± 1.25	**0.008**	10.93 ± 1.42	10.52 ± 1.45	**0.132**	11.30 ± 1.26	11.12 ± 1.43	0.489
EPA 20:5n-3	0.34 ± 0.15	0.35 ± 0.17	0.721	0.40 ± 0.14	0.99 ± 0.71	**<0.001**	0.40 ± 0.13	0.86 ± 0.67	**<0.001**	0.39 ± 0.11	0.72 ± 0.56	**<0.001**
ObA 22:5n-6	0.45 ± 0.12	0.47 ± 0.11	0.245	0.46 ± 0.21	0.36 ± 0.14	**0.001**	0.43 ± 0.09	0.34 ± 0.12	**<0.001**	0.43 ± 0.09	0.38 ± 0.12	**0.034**
DPA 22:5n-3	1.23 ± 0.22	1.23 ± 0.19	0.912	1.29 ± 0.26	1.61 ± 0.41	**<0.001**	1.30 ± 0.23	1.52 ± 0.34	**<0.001**	1.30 ± 0.18	1.46 ± 0.30	**0.003**
DHA 22:6n-3	2.56 ± 0.49	2.49 ± 0.41	0.364	2.49 ±0.52	3.18 ± 0.78	**<0.001**	2.71 ± 0.55	3.35 ± 0.87	**<0.001**	2.69 ± 0.55	3.05 ± 0.76	**0.008**
O3I	3.75 ± 0.60	3.68 ± 0.51	0.499	3.74 ± 0.65	5.09 ± 1.49	**<0.001**	3.96 ± 0.68	5.13 ± 1.56	**<0.001**	3.95 ± 0.64	4.67 ± 1.32	**0.001**

AA = arachidonic acid, DHA = docosahexaenoic acid, DPA= docosapentaenoic acid, EPA = eicosapentaenoic acid, O3I = Omega-3 Index, ObA = Osbond acid

Supplemental Table S3: Multilevel analyses of cognitive test scores predicted by condition and Omega-3 Index, participant whom participated in all test moments

			Estimate (SE)	95%CI		Estimate (SE)	95%CI
D2- Total	Test moment	T2	45.10 (3.72)	**[37.90; 52.30]**	T2	47.13 (3.10)	**[41.12; 53.14]**
		T3	87.65 (3.81)	**[80.28; 95.02]**	T3	84.45 (3.07)	**[78.51; 90.39]**
	Condition	Krill	-4.78 (8.85)	[-21.96; 12.39]	O3I	-0.12 (1.63)	[-3.28; 3.03]
	Interaction	T2 × krill	4.24 (5.43)	[-6.25; 14.73]			
		T3 × krill	-7.18 (5.45)	[-17.71; 3.36]			
D2- Correct	Test moment	T2	21.68 (1.61)	**[18.57; 24.79]**	T2	22.74 (1.35)	**[20.13;25.34]**
		T3	42.98 (1.61)	**[39.79; 46.17]**	T3	41.47 (1.33)	**[38.89;44.05]**
	Condition[1]	Krill	0.30 (4.11)	[-7.67; 8.28]	O3I	-0.52 (0.71)	[-1.90;0.86]
	Interaction[2]	T2 × krill	1.31 (2.34)	[-3.23; 5.84]			
		T3 × krill	-4.01 (2.36)	[-8.57; 0.54]			
D2- F1	Test moment	T2	-0.16 (0.04)	**[-0.25; -0.07]**	T2	-0.16 (0.04)	**[-0.23; -0.08]**
		T3	-0.25 (0.05)	**[-0.34; -0.16]**	T3	-0.27 (0.04)	**[-0.34; -0.20]**
	Condition	Krill	-0.15 (0.11)	[-0.37; 0.07]	O3I	0.02 (0.02)	[-0.02; 0.06]
	Interaction	T2 × krill	0.04 (0.07)	[-0.10; 0.17]			
		T3 × krill	-0.03 (0.07)	[-0.16; 0.11]			
D2-F2	Test moment	T2	-0.14 (0.13)	[-0.39; 0.12]	T2	-0.03 (0.11)	[-0.23; 0.18]
		T3	-0.46 (0.15)	**[-0.73; -0.18]**	T3	-0.48 (0.13)	**[-0.76; -0.26]**
	Condition	Krill	-0.24 (0.18)	[-0.59; 0.11]	O3I	-0.04 (0.05)	[-0.14; 0.06]
	Interaction	T2 × krill	0.19 (0.21)	[-0.21; 0.58]			
		T3 × krill	-0.03 (0.23)	[-0.52; 0.41]			
LDST	Test moment	T2	2.27 (0.50)	**[1.30; 3.24]**	T2	2.43 (0.41)	[1.64; 3.22]
		T3	4.64 (0.51)	**[3.65; 5.63]**	T3	4.58 (0.40)	[3.80; 5.36]
	Condition	Krill	-0.61 (0.87)	[-2.29; 1.07]	O3I	-0.07 (0.20)	[-0.47; 0.33]
	Interaction	T2 × krill	0.20 (0.73)	[-1.22; 1.62]			
		T3 × krill	-0.06 (0.73)	[-1.48; 1.36]			
Shifting	Test moment	T2	-1.72 (0.86)	**[-3.38; -0.06]**	T2	-1.90 (0.68)	**[-3.22; -0.59]**
		T3	- 1.29 (0.87)	[-2.97; 0.40]	T3	-1.95 (0.68)	**[-3.26; -0.64]**
	Condition	Krill	-0.10 (0.98)	[-1.99; 1.80]	O3I	0.15 (0.28)	[-0.39; 0.68]
	Interaction	T2 × krill	-0.11(1.26)	[-2.54; 2.32]			
		T3 × krill	-1.40 (1.26)	[-3.85; 1.04]			
Interference	Test moment	T2	-3.82 (0.76)	**[-5.29; -2.35]**	T2	-3.39 (0.61)	**[-4.58; -2.21]**
		T3	-5.25 (0.77)	**[-6.75; -3.76]**	T3	-5.38 (0.61)	**[-6.55; -4.20]**
	Condition	Krill	-0.40 (1.05)	[-2.44; 1.65]	O3I	-0.01 (0.29)	[-0.57; 0.54]
	Interaction	T2 × krill	0.87 (1.11)	[-1.28; 3.02]			
		T3 × krill	-0.45 (1.11)	[-2.60; 1.70]			

D2 = D2 test of attention, D2 correct = number of target stimuli processed, D2F1 = errors of omission on D2, D2F2 = errors of commission on D2, LDST = letter digit substitution task, O3I = Omega-3 Index, T2 = test moment 6 months, T3 = test moment 12 months. All models included test moment, condition (krill/placebo) plus covariates (alcohol consumption, smoking behaviour, age at baseline, puberty status at baseline, BMI, highest level of parental education, sex, cohort number and diagnosis). The factor krill indicates the score of somebody in the krill oil group compared to the placebo group. Significant 95 % CI are printed in bold. The model with condition also includes the time moment X condition interaction factor. Note that a lower score on D2F1, D2F2, Shifting and Interference is a better performance.

Supplementary Table S4: Multilevel analyses of cognitive test scores predicted by condition (intention to treat) and according to Omega-3 Index for cohort 1

**Cohort 1**			Estimate (SE)	95%CI		Estimate (SE)	95%CI
D2- Total	Test moment	T2	45.69 (4.93)	**[36.28; 55.10]**	T2	45.08 (4.32)	**[36.80; 53.35]**
		T3	90.33 (5.11)	**[80.56; 100.09]**	T3	85.33 (4.39)	**[76.92; 93.74]**
	Condition	Krill	0.68 (12.23)	[-22.84; 24.19]	O3I	-1.42 (2.34)	[-5.90; 3.06]
	Interaction	T2 × krill	-3.06 (7.91)	[-18.18; 12.06]			
		T3 × krill	-15.34 (8.08)	[-30.77; 0.09]			
D2- Correct	Test moment	T2	22.05 (2.22)	**[17.82; 26.29]**	T2	22.24 (1.94)	**[18.53; 25.96]**
		T3	43.78 (2.30)	**[39.39; 48.18]**	T3	41.99 (1.97)	**[38.22; 45.77]**
	Condition	Krill	-0.75 (5.61)	[-11.53; 10.03]	O3I	-1.05 (1.05)	[-3.06; 0.96]
	Interaction	T2 × krill	-0.77 (3.56)	[-7.57; 6.03]			
		T3 × krill	-6.42 (3.64)	[-13.36; 0.53]			
D2- F1	Test moment	T2	-0.18 (0.06)	**[-0.31; -0.04]**	T2	-0.21 (0.06)	**[-0.31; -0.10]**
		T3	-0.29 (0.07)	**[-0.41; -0.14]**	T3	-0.35 (0.06)	**[-0.47; -0.24]**
	Condition	Krill	-0.12 (0.16)	[-0.19; 0.44]	O3I	-0.02 (0.03)	[-0.09; 0.03]
	Interaction	T2 × krill	-0.07 (0.10)	[-0.28; 0.14]			
		T3 × krill	-0.15 (0.11)	[-0.37; 0.04]			
D2-F2	Test moment	T2	-0.01 (0.17)	[-0.37; 0.34]	T2	-0.12 (0.15)	[-0.38; 0.20]
		T3	-0.24 (0.19)	[-0.63; 0.16]	T3	-0.37 (0.17)	**[-0.67; -0.05]**
	Condition	Krill	-0.04 (0.26)	[-0.53; 0.49]	O3I	-0.004 (0.08)	[-0.16; 0.14]
	Interaction	T2 × krill	-0.35 (0.30)	[-0.93; 0.23]			
		T3 × krill	-0.27 (0.33)	[-0.96; 0.30]			
LDST	Test moment	T2	3.13 (0.70)	**[1.78; 4.48]**	T2	2.89 (0.61)	**[1.73; 4.05]**
		T3	4.22 (0.73)	**[2.83; 5.61]**	T3	4.40 (0.61)	**[3.23; 5.58]**
	Condition	Krill	-0.22 (1.29)	[-2.70; 2.25]	O3I	-0.004 (0.31)	[-0.61; 0.60]
	Interaction	T2 × krill	-0.61 (1.13)	[-2.77; 1.55]			
		T3 × krill	0.79 (1.15)	[-1.42; 2.99]			
Shifting	Test moment	T2	-1.66 (1.21)	[-3.98; 0.66]	T2	-2.09 (1.01)	**[-4.02; -0.16]**
		T3	-1.94 (1.23)	[-4.31; 0.42]	T3	-2.27 (1.02)	**[-4.23; -0.30]**
	Condition	Krill	-0.83 (1.40)	[-3.53; 1.87]	O3I	0.23 (0.41)	[-0.57; 1.02]
	Interaction	T2 × krill	-0.69 (1.95)	[-4.43; 3.05]			
		T3 × krill	-1.05 (1.98)	[-4.85; 2.74]			
Interference	Test moment	T2	-4.12 (1.11)	**[-6.25; -1.99]**	T2	-3.79 (0.95)	**[-5.57; -1.95]**
		T3	-5.15 (1.14)	**[-7.33; -2.96]**	T3	-5.08 (0.96)	**[-6.91; -3.24]**
	Condition	Krill	-1.01 (1.67)	[-4.21; 2.19]	O3I	0.16 (0.46)	[-0.72; 1.05]
	Interaction	T2 × krill	1.22 (1.79)	[-2.20; 4.63]			
		T3 × krill	-0.03 (1.82)	[-3.51; 3.45]			

D2 = D2 test of attention, D2 correct = number of target stimuli processed, D2F1 = errors of omission on D2, D2F2 = errors of commission on D2, LDST = letter digit substitution task, O3I = Omega-3 Index, T2 = test moment 6 months, T3 = test moment 12 months. All models included test moment, condition (krill/placebo) plus covariates (alcohol consumption, smoking behaviour, age at baseline, puberty status at baseline, BMI, highest level of parental education, sex, cohort number and diagnosis). Significant 95 % CI are printed in bold. The model with condition also includes the time moment X condition interaction factor. Note that a lower score on D2F1, D2F2, Shifting and Interference is a better performance.

Supplementary Table S5: Multilevel analyses of cognitive test scores predicted by condition (intention to treat) and according to Omega-3 Index for cohort 2

Cohort 2			Estimate (SE)	95%CI		Estimate (SE)	95%CI
D2- Total	Test moment	T2	52.93 (5.32)	**[42.71; 63.16]**	T2	51.36 (4.22)	**[43.22; 59.50]**
		T3	89.45 (5.78)	**[78.33; 100.57]**	T3	85.88 (4.32)	**[77.54; 94.22]**
	Condition	Krill	-10.97 (10.65)	[-31.54; 9.61]	O3I	0.43 (2.26)	[-3.93; 4.80]
	Interaction	T2 × krill	-2.15 (7.26)	[-16.10; 11.80]			
		T3 × krill	-6.30 (7.68)	[-21.07; 8.48]			
D2- Correct	Test moment	T2	24.57 (2.22)	**[20.30; 28.84]**	T2	23.99 (1.77)	**[20.57; 27.41]**
		T3	43.90 (2.42)	**[39.25; 48.55]**	T3	41.79 (1.82)	**[38.28; 45.29]**
	Condition	Krill	-1.07 (4.91)	[-10.56; 8.42]	O3I	-0.15 (0.96)	[-2.00;1.71]
	Interaction	T2 × krill	-1.35 (3.03)	[-7.18; 4.48]			
		T3 × krill	-4.04 (3.21)	[-10.21; 2.14]			
D2- F1	Test moment	T2	-0.08 (0.06)	[-0.19; 0.04]	T2	-0.10 (0.05)	**[-0.20; -0.004]**
		T3	-0.19 (0.06)	**[-0.31; -0.06]**	T3	-0.20 (0.05)	**[-0.31; -0.11]**
	Condition	Krill	-0.26 (0.13)	[-0.56; 0.008]	O3I	0.03 (0.03)	[-0.03; 0.08]
	Interaction	T2 × krill	0.02 (0.08)	[-0.15; 0.17]			
		T3 × krill	-0.01 (0.09)	[-0.20; 0.14]			
D2-F2	Test moment	T2	-0.48 (0.19)	**[-0.83; -0.14]**	T2	-0.10 (0.14)	[-0.39; 0.14]
		T3	-0.95 (0.24)	**[-1.44; -0.49]**	T3	-0.71 (0.17)	**[-1.07; -0.38]**
	Condition	Krill	-0.41 (0.19)	**[-0.79; -0.05]**	O3I	-0.08 (0.07)	[-0.24; 0.04]
	Interaction	T2 × krill	0.58 (0.26)	**[0.09; 1.06]**			
		T3 × krill	0.34 (0.33)	[-0.34; 0.96]			
LDST	Test moment	T2	1.81 (0.67)	**[0.53; 3.09]**	T2	2.11 (0.52)	**[1.11; 3.12]**
		T3	5.42 (0.72)	**[4.03; 6.81]**	T3	4.79 (0.54)	**[3.76; 5.82]**
	Condition	Krill	0.05 (1.02)	[-1.91; 2.02]	O3I	-0.12 (0.27)	[-0.63; 0.40]
	Interaction	T2 × krill	0.37 (0.91)	[-1.38; 2.12]			
		T3 × krill	-1.16 (0.96)	[-3.00; 0.69]			
Shifting	Test moment	T2	-1.63 (1.11)	[-3.77; 0.51]	T2	-1.59 (0.83)	[-3.21; 0.02]
		T3	-0.59 (1.20)	[-2.89; 1.71]	T3	-1.57 (0.86)	[-3.24; 0.10]
	Condition	Krill	0.30 (1.19)	[-2.00; 2.60]	O3I	-0.009 (0.36)	[-0.71; 0.69]
	Interaction	T2 × krill	0.02 (1.51)	[-2.90; 2.93]			
		T3 × krill	-1.86 (1.59)	[-4.93; 1.21]			
Interference	Test moment	T2	-3.89 (0.94)	**[-5.70 -2.07]**	T2	-3.09 (0.72)	**[-4.49; -1.69]**
		T3	-5.68 (1.02)	**[-7.64; -3.72]**	T3	-5.45 (0.74)	**[-6.89; -4.02]**
	Condition	Krill	-1.62 (1.18)	[-3.90; 0.66]	O3I	-0.17 (0.35)	[-0.84; 0.50]
	Interaction	T2 × krill	1.15 (1.29)	[-1.34; 3.63]			
		T3 × krill	0.16 (1.35)	[-2.45; 2.76]			

D2 = D2 test of attention, D2 correct = number of target stimuli processed, D2F1 = errors of omission on D2, D2F2 = errors of commission on D2, LDST = letter digit substitution task, O3I = Omega-3 Index, T2 = test moment 6 months, T3 = test moment 12 months. All models included test moment, condition (krill/placebo) plus covariates (alcohol consumption, smoking behaviour, age at baseline, puberty status at baseline, BMI, highest level of parental education, sex, cohort number and diagnosis). Significant 95 % CI are printed in bold. The model with condition also includes the time moment X condition interaction factor. Note that a lower score on D2F1, D2F2, Shifting and Interference is a better performance.

Supplemental Table S6: Multilevel analyses of DSF and DSB predicted by condition and Omega-3 Index, participant whom participated in all test moments

		Odds ratio (SE)	95%CI		Odds ratio (SE)	95%CI
Digit Span forward						
Test moment	T2	0.51 (1.34)	**[0.29; 0.90]**	T2	0.58 (1.26)	**[0.37; 0.92]**
	T3	1.28 (1.34)	[0.72; 2.27]	T3	1.25 (1.26)	[0.80; 1.96]
Condition	Krill	1.03 (1.46)	[0.49; 2.17]	O3I	1.03 (1.11)	[0.84; 1.27]
Interaction	T2 × krill	1.46 (1.53)	[0.64; 3.37]			
	T3 × krill	1.06 (1.53)	[0.46; 2.44]			
Digit Span backward						
Test moment	T2	1.31 (1.32)	[0.76; 2.24]	T2	1.44 (1.24)	[0.94; 2.21]
	T3	1.53 (1.33)	[0.87; 2.69]	T3	1.58 (1.25)	**[1.02; 2.43]**
Condition	Krill	0.81 (1.41)	[0.41; 1.59]	O3I	0.99 (1.10)	[0.82; 1.20]
Interaction	T2 × krill	1.15 (1.49)	[0.52; 2.51]			
	T3 × krill	1.02 (1.51)	[0.46; 2.29]			

O3I = Omega-3 Index, T2 = test moment 6 months, T3 = test moment 12 months. All models included test moment, condition (krill/placebo) or Omgea-3 Index plus covariates (alcohol consumption, smoking behaviour, age at baseline, puberty status at baseline, BMI, highest level of parental education, sex, cohort number and diagnosis). The model with condition also includes the time moment X condition interaction factor. Please note that for odds ratios significance is indicated by a 95% CI which does not contain 1.

Supplementary Table S7: Multilevel analyses of digit span forward and digit span backward, predicted by either condition (intention to treat) or Omega-3 Index for Cohort 1

Cohort 1		Odds ratio (SE)	95%CI		Odds ratio (SE)	95%CI
Digit Span forward						
Test moment	T2	0.41 (1.48)	**[0.19; 0.89]**	T2	0.47 (1.39)	**[0.25; 0.91]**
	T3	1.31 (1.49)	[0.60; 2.89]	T3	0.85 (1.40)	[0.44; 1.64]
Condition	Krill	1.52 (1.77)	[0.49; 4.65]	O3I	0.95 (1.18)	[0.69; 1.30]
Interaction	T2 × krill	1.49 (1.86)	[0.44; 5.02]			
	T3 × krill	0.36 (1.92)	[0.10; 1.30]			
Digit Span backward						
Test moment	T2	1.33 (1.44)	[0.65; 2.72]	T2	1.04 (1.35)	[0.57; 1.89]
	T3	1.21 (1.47)	[0.57; 2.55]	T3	1.28 (1.37)	[0.69; 2.38]
Condition	Krill	0.92 (1.65)	[0.35; 2.44]	O3I	1.04 (1.15)	[0.79; 1.38]
Interaction	T2 × krill	0.61 (1.79)	[0.20; 1.92]			
	T3 × krill	1.13 (1.84)	[0.34; 3.73]			

O3I = Omega-3 Index, T2 = test moment 6 months, T3 = test moment 12 months. All models included test moment, condition (krill/placebo) or Omgea-3 Index plus covariates (alcohol consumption, smoking behaviour, age at baseline, puberty status at baseline, BMI, highest level of parental education, sex, cohort number and diagnosis). The model with condition also includes the time moment X condition interaction factor. Please note that for odds ratios significance is indicated by a 95% CI which does not contain 1.

Supplementary Table S8: Multilevel analyses of digit span forward and digit span backward, predicted by either condition (intention to treat) or Omega-3 Index for Cohort 2

Cohort 2		Odds ratio (SE)	95%CI		Odds ratio (SE)	95%CI
Digit Span forward						
Test moment	T2	0.47 (1.49)	[0.22; 1.03]	T2	0.60 (1.35)	[0.33;1.09]
	T3	1.10 (1.52)	[0.49; 2.49]	T3	1.56 (1.35)	[0.87; 2.82]
Condition	Krill	0.70 (1.55)	[0.30; 1.65]	O3I	1.15 (1.14)	[0.88; 1.50]
Interaction	T2 × krill	2.05 (1.73)	[0.70; 6.00]			
	T3 × krill	2.24 (1.75)	[0.75; 6.68]			
Digit Span backward						
Test moment	T2	1.45 (1.46)	[0.69; 3.02]	T2	2.15 (1.33)	**[1.24; 3.75]**
	T3	2.32 (1.51)	**[1.03; 5.21]**	T3	2.12 (1.35)	**[1.18; 3.81]**
Condition	Krill	0.87 (1.51)	[0.39; 1.96]	O3I	0.91 (1.14)	[0.71; 1.17]
Interaction	T2 × krill	1.66 (1.66)	[0.61; 4.50]			
	T3 × krill	0.76 (1.73)	[0.26; 2.23]			

O3I = Omega-3 Index, T2 = test moment 6 months, T3 = test moment 12 months. All models included test moment, condition (krill/placebo) or Omgea-3 Index plus covariates (alcohol consumption, smoking behaviour, age at baseline, puberty status at baseline, BMI, highest level of parental education, sex, cohort number and diagnosis). The model with condition also includes the time moment X condition interaction factor. Please note that for odds ratios significance is indicated by a 95% CI which does not contain 1.
